# Inflammatory landscape of *Gardnerella vaginalis*-associated bacterial vaginosis in rats: an integrated multi-omics analysis

**DOI:** 10.3389/fimmu.2026.1914434

**Published:** 2026-09-22

**Authors:** Xiao-Feng Ruan, Xiao-Meng Xue, Gao-Pi Deng, Hao-Meng Wu, Si Chen, Xiang-Dan Hu, Fang-Fang Zhu, Yun-Yun Luo, Dan-Ting Wen

**Affiliations:** 1 Guangzhou University of Chinese Medicine, Guangzhou, China; 2 The Second Affiliated Hospital of Guangzhou University of Chinese Medicine, Guangzhou, China; 3 The First Clinical Medical School of Guangzhou University of Chinese Medicine, Guangzhou, China; 4 Lingnan Medical Research Center of Guangzhou University of Chinese Medicine, Guangzhou, China; 5 The First Affiliated Hospital of Guangzhou University of Chinese Medicine, Guangzhou, China; 6 State Key Laboratory of Traditional Chinese Medicine Syndrome, Guangzhou, China

**Keywords:** arachidonic acid metabolism, bacterial vaginosis, *Gardnerella vaginalis*, immunometabolism, metabolomics, tryptophan metabolism, vaginal microbiome

## Abstract

**Background:**

Bacterial vaginosis (BV) is a prevalent vaginal dysbiosis primarily associated with *Gardnerella vaginalis* (*G. vaginalis*). However, its pathogenic mechanisms remain incompletely understood, particularly the interplay between microbial dysbiosis and host metabolic perturbations. In this study, a *G. vaginalis*-associated BV-like rat model was established, and integrated multi-omics analysis was performed to characterize its inflammatory landscape.

**Methods:**

Female Sprague-Dawley rats received antibiotic pretreatment followed by intravaginal inoculation with *G. vaginalis* to establish a BV-like model. Gram staining, histological examination, and serum cytokine enzyme-linked immunosorbent assay (ELISA) evaluated the model phenotype. 16S rRNA gene sequencing characterized the vaginal microbiota, and UHPLC–MS/MS profiled the vaginal metabolome. ELISA quantified serum metabolites. Western blotting (WB) assessed proteins related to Toll-like receptor (TLR)/nuclear factor-kappa B (NF-κB) signaling in uterine tissues.

**Results:**

The BV-like phenotype was characterized by clue cell-like epithelial cells, histopathological injury, and an altered serum cytokine profile. 16S sequencing revealed nonsignificant trends toward increased diversity and reduced evenness in the model group. Although overall microbial community composition did not differ significantly between groups, within-group dispersion was significantly lower in the model group. The genera *Gardnerella*, *Staphylococcus*, *Bacteroides*, and *Aerococcus* were overrepresented, whereas *Collinsella* was underrepresented; among these genera only *Aerococcus* remained significant after FDR correction. Untargeted vaginal metabolomics and targeted serum measurements indicated enhanced arachidonic acid (AA)-related metabolism and compartment-specific remodeling of tryptophan (TRP) metabolism. Group-adjusted partial correlation analysis identified nominal positive correlations of *Gardnerella* with AA and *Collinsella* with 5-hydroxyindole-3-acetic acid (5-HIAA), as well as a nominal negative correlation of *Blastococcus* with thromboxane B_2_ (TXB_2_); however, none remained significant after FDR correction. WB revealed increased abundance of uterine proteins associated with TLR2/4–NF-κB–COX-2 inflammatory signaling in the model group.

**Conclusions:**

These findings suggest that the *G. vaginalis*-associated BV-like phenotype is linked to vaginal microbial alterations, enhanced AA-related metabolism, compartment-specific remodeling of TRP metabolism, and upregulation of proteins related to uterine TLR/NF-κB inflammatory signaling. This microbiome–metabolome–immune network may help explain how *G. vaginalis*-associated vaginal dysbiosis contributes to BV-like inflammatory changes and may be related to endometrial inflammation.

## 1 Introduction

Bacterial vaginosis (BV) is a common vaginal dysbiosis characterized by a shift in the vaginal microbiota from a *Lactobacillus*-dominant community to an overgrowth of anaerobic and facultative anaerobic bacteria, most notably *Gardnerella vaginalis* (*G. vaginalis*) (1). Clinically, this condition often manifests as malodor and discharge, but can remain asymptomatic (2). Beyond its immediate symptoms, BV is associated with serious reproductive health complications, including adverse pregnancy outcomes such as preterm birth and spontaneous abortion; an elevated risk of acquiring HIV and other sexually transmitted infections (STIs); and an increased susceptibility to urogenital infections and pelvic inflammatory disease (PID) (3). With an estimated prevalence of 26% in the general population, BV constitutes a global public health concern, imposing a substantial economic burden attributable to both its direct management and the costs linked to treating its clinical sequelae (4).

*G. vaginalis* has been recognized as the cornerstone pathogen in BV, playing a pivotal role beyond simple colonization (5). *G. vaginalis* is a facultatively anaerobic, non-spore-forming, Gram-variable pleomorphic coccobacillus characterized by a thin peptidoglycan cell wall (6). Clinically, the extensive adhesion of *G. vaginalis* to vaginal epithelial cells and subsequent biofilm formation are manifested by the presence of “clue cells”, a clinical hallmark of BV (7, 8). Our previous clinical studies have highlighted the potential adverse impact of *G. vaginalis* enrichment, demonstrating that vaginal microbiota disruption characterized by its enrichment is significantly associated with severe upper reproductive tract complications (9, 10). However, the mechanisms by which *G. vaginalis* contributes to BV progression and subsequent reproductive complications remain incompletely elucidated. Current evidence suggests that its pathogenic potential is not driven by a single virulence factor (11). Instead, its pathogenesis relies on a multifaceted cascade that begins with epithelial adhesion and biofilm formation (12, 13), subsequently extending to vaginolysin-mediated epithelial cytotoxicity (14), local microenvironmental remodeling (15), Toll-like receptor (TLR)-mediated immune modulation (16, 17), and synergistic metabolic interactions with other BV-associated anaerobes (18). Therefore, establishing a reliable *in vivo* model to explore the inflammatory landscape of the vaginal microenvironment from the perspective of host–microbiome–metabolome interactions is crucial for further understanding the pathogenic mechanisms of BV.

In this study, we established a *G. vaginalis*-associated BV-like rat model through antibiotic pretreatment followed by repeated intravaginal *G. vaginalis* inoculation, which reproduced several key clinical features of the disease. This model served as a platform to systematically investigate the interplay among the vaginal microbiome, metabolome, and host inflammatory signaling. Specifically, we assessed the vaginal microbiota via 16S rRNA sequencing and profiled the vaginal metabolome using untargeted ultra-high-performance liquid chromatography coupled with tandem mass spectrometry (UHPLC–MS/MS). This strategy allowed us to characterize alterations across biological scales—from the vaginal microbiota and metabolic profiles to host inflammatory responses. Furthermore, we evaluated the expression of uterine TLR/NF-κB pathway-related proteins to explore a potential relationship with upper reproductive tract complications. Ultimately, through this integrated approach, we aimed to characterize how *G. vaginalis*-associated BV reshapes the vaginal microbiota and metabolic microenvironment, and to explore potential relationships between these alterations and endometrial inflammation reflected in host inflammatory signaling.

## 2 Materials and methods

### 2.1 *Gardnerella vaginalis* inoculum preparation

*G. vaginalis* (ATCC 14018) was obtained from the Guangdong Microbial Culture Collection Center (GDMCC, Guangdong, China). *G. vaginalis* was cultivated in brain heart infusion (BHI) medium (Huankai Microbial Co., Ltd., Guangdong, China) supplemented with 10% fetal bovine serum (FBS) (Huankai Microbial Co., Ltd.) at 37 °C for 48 h under 5% CO_2_ environment. *G. vaginalis* was suspended in saline at a concentration of 1 × 10^9^ CFU/mL.

### 2.2 *Gardnerella vaginalis*-associated bacterial vaginosis-like rat model establishment and sample collection

Female Sprague-Dawley rats (N = 16, specific pathogen-free (SPF) grade, 12 weeks old, weight 316.5 ± 13.0 g) were obtained from Guangdong Medical Laboratory Animal Center (Guangdong, China). All rats were maintained on a 12-h light and dark cycle at an appropriate temperature (22 ± 2 °C) and humidity (50% ± 10%) with free access to food and water.

During the 1-week acclimation period, estrous-cycle stage was assessed by vaginal smear. Rats in diestrus were enrolled and assigned to the model group (N = 8) or the control group (N = 8) by simple randomization using a random-number table. This procedure matched the groups for estrous stage at baseline and reduced initial variation associated with endogenous hormonal status. No formal *a priori* sample-size calculation was performed. Eight rats per group were included to provide independent biological replication within the practical constraints of this exploratory multi-omics animal study. For the model group, rats received intravaginal antibiotic pretreatment (120 μL of a mixture of 100 mg/mL penicillin and 100 mg/mL gentamicin) for 6 consecutive days (days 1 to 6). Then, the rats were inoculated with 120 μL of a 1 × 10^9^ CFU/mL *G. vaginalis* suspension into the vagina to a depth of 5 mm for 28 consecutive days (days 7 to 34) to generate a *G. vaginalis*-associated BV-like vaginal dysbiosis model. For the control group, rats received saline instead of the antibiotic pretreatment and *G. vaginalis* suspension. During vaginal injection, rats were kept in a supine position under 4% isoflurane anesthesia to reduce reflux toward the uterine cavity (**Figure 1A**).

**Figure 1:**
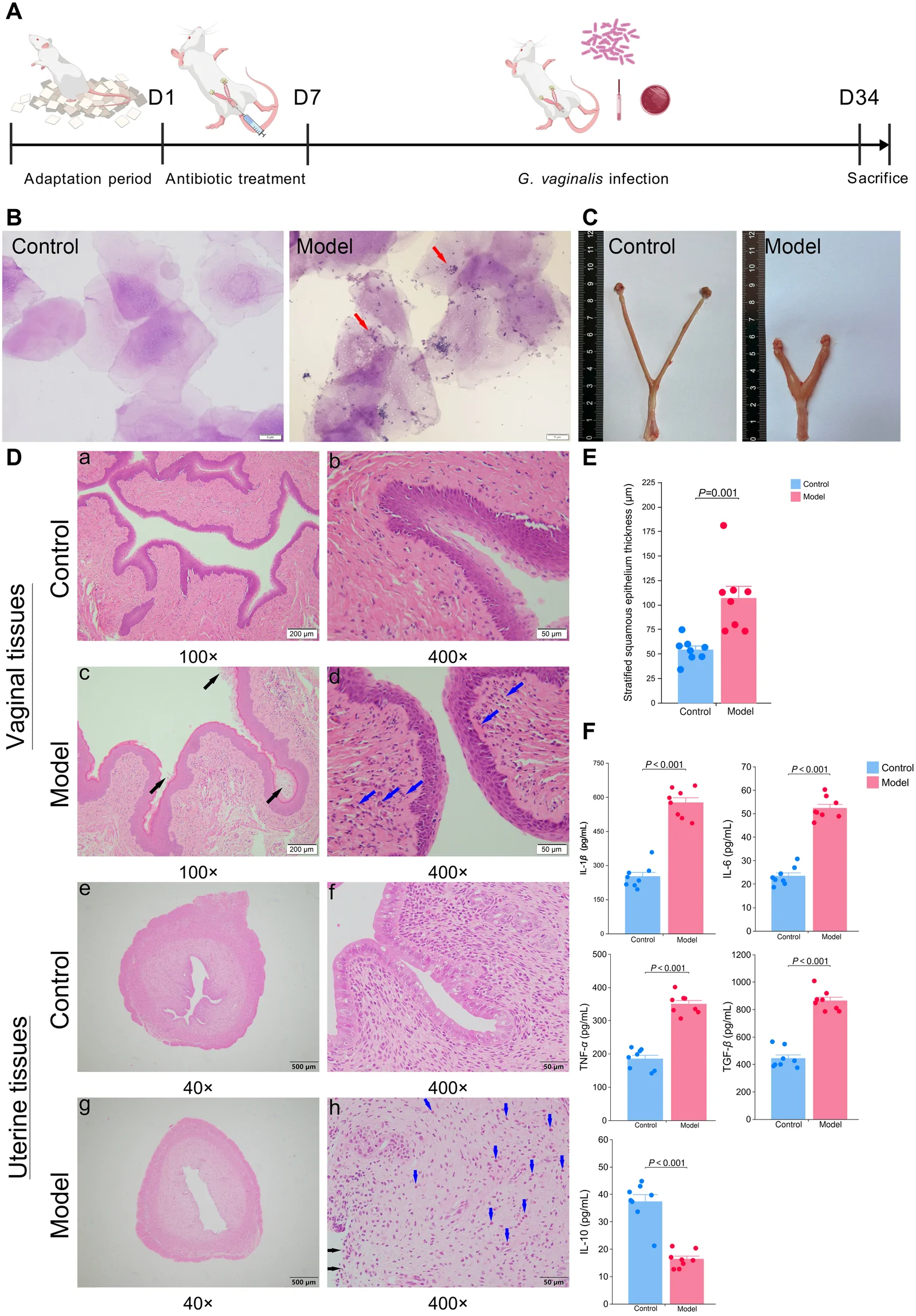
Characterization of the *Gardnerella vaginalis*-associated bacterial vaginosis-like rat model. **(A)** Experimental scheme for model establishment. **(B)** Representative Gram-stained vaginal smears. The red arrows indicate clue cell-like epithelial cells. Scale bar = 5 μm; magnification × 1000. **(C)** Gross morphology of the excised reproductive tracts. **(D)** Representative histology of vaginal and uterine tissue sections stained with hematoxylin and eosin (H&E). **(a)** Vaginal tissues in the control group. Scale bar = 200 μm; magnification × 100. **(b)** Vaginal tissues in the control group. Scale bar = 50 μm; magnification × 400. **(c)** Vaginal tissues in the model group. Scale bar = 200 μm; magnification × 100. The black arrows indicate damaged vaginal epithelium and the exfoliated vaginal epithelial cells. **(d)** Vaginal tissues in the model group. Scale bar = 50 μm; magnification × 400. The blue arrows indicate inflammatory cell infiltration in the vaginal mucosa and lamina propria. **(e)** Uterine tissues in the control group. Scale bar = 500 μm; magnification × 40. **(f)** Uterine tissues in the control group. Scale bar = 50 μm; magnification × 400. **(g)** Uterine tissues in the model group. Scale bar = 500 μm; magnification × 40. **(h)** Uterine tissues in the model group. Scale bar = 50 μm; magnification × 400. The black arrows indicate damaged endometrial epithelial cells, and the blue arrows indicate inflammatory cells infiltrating the uterine mucosal layers. **(E)** Quantitative analysis of stratified squamous epithelium thickness. **(F)** Serum concentrations of interleukin-1 beta (IL-1*β*), IL-6, tumor necrosis factor-alpha (TNF-*α*), transforming growth factor-beta (TGF-*β*) and IL-10. Data are presented as the means ( $\bar{X}$) ± standard error of the mean (SEM); n = 8 rats per group. Statistical significance was determined using Student’s *t-tests*.

On day 35 of the experiment, the rats were deeply anesthetized with inhaled isoflurane at 4% for induction and 2% for maintenance, and samples were collected. Vaginal secretions were collected using three sterile swabs. While one swab was gently smeared on a glass slide for Gram staining, the other two swabs were immediately frozen in liquid nitrogen and then stored at -80 °C until DNA extraction and metabolomics analysis. After confirmation of adequate anesthetic depth, blood was collected from the abdominal aorta into tubes without heparin, and serum was subsequently separated. Following terminal blood collection, euthanasia was completed by exsanguination under deep isoflurane anesthesia, and death was confirmed before tissue collection. The reproductive tract of rats (vagina, uterus, fallopian tubes, and ovaries) was dissected, gently washed in saline to remove blood, and subjected to subsequent histological examinations and protein detections.

Our study was approved by the Laboratory Animal Ethics Committee of Guangdong Medical Laboratory Animal Center [ethical approval no. B202311-9].

### 2.3 Gram staining and histological examinations

Vaginal smears were air-dried, heat-fixed, and then stained using a Gram Stain Kit (Hunan BKMAM Holding Co., Ltd., Hunan, China). Slides were observed under a 100× oil-immersion objective (Olympus, Tokyo, Japan).

The gross morphology of the excised reproductive tracts was photographed and assessed.

The vaginal and uterine tissues were fixed in 4% paraformaldehyde, embedded in paraffin, sectioned at a thickness of 5 μm, stained with hematoxylin and eosin (H&E), and observed under an optical microscope (Olympus). The thickness of the vaginal keratinized stratified squamous epithelium was quantified through digital image quantification using the cellSens Dimension imaging software (Olympus), based on the mean of three randomly selected fields for each animal. Histopathological assessment and epithelial-thickness measurements were performed with group allocation masked.

### 2.4 ELISA detection

Enzyme-linked immunosorbent assay (ELISA) was applied to analyze serum levels of cytokines and metabolites. Serum samples in rats were detected using ELISA kits (Jiangsu Meimian Industrial Co., Ltd., Jiangsu, China) following the manufacturer’s protocols. The measured cytokines included interleukin-1 beta (IL-1*β*), IL-6, IL-10, tumor necrosis factor-alpha (TNF-*α*), and transforming growth factor-beta (TGF-*β*). The assessed arachidonic acid (AA) mediators included free AA, 6-keto-prostaglandin F1 alpha (6-keto-PGF_1α_), thromboxane B_2_ (TXB_2_), leukotriene B_4_ (LTB_4_), and prostaglandin E_2_ (PGE_2_). The tryptophan (TRP)-related analytes included TRP, kynurenine (KYN), and kynurenic acid (KYNA) (**Supplementary Table 1**). Each sample was analyzed in triplicate, and the mean of the technical replicates was used for statistical analysis. The KYN-to-TRP ratio (KYN/TRP) was calculated after converting KYN from pg/mL to ng/mL, and then the ratio was reported on a ×10^3^ scale.

### 2.5 16S rRNA sequencing

Vaginal secretions from rats were subjected to 16S rRNA gene sequencing. Vaginal bacterial genomic DNA was extracted by the cetyltrimethyl ammonium bromide method. The V3-V4 regions were amplified using specific primers (341F and 806R) with the barcode by a polymerase chain reaction (PCR) assay (Phusion^®^ High-Fidelity PCR Master Mix, New England Biolabs, Ipswich, MA, USA). The sequences for the primers are as follows: 341F: CCTAYGGGRBGCASCAG, and 806R: GGACTACNNGGGTATCTAAT. The amplified PCR products were identified using agarose gel electrophoresis and purified with AMPure XP beads (Beckman Coulter, CA, USA). Libraries were constructed by the NEBNext^®^ Ultra™ II DNA Library Prep Kit (New England Biolabs), and the concentrations were evaluated by the Qubit^®^ 2.0 Fluorometer (Thermo Fisher Scientific, MA, USA). The quantified libraries were then pooled and sequenced by NovaSeq6000 (Illumina, San Diego, CA, USA). The raw tags were obtained by splicing the reads using FLASH (version 1.2.11) and then filtered to acquire high-quality clean tags using FASTP (version 0.23.1). The clean tags were compared to the Silva database (https://www.arb-silva.de/) to identify chimera sequences, which were subsequently removed using the vsearch package (version 2.16.0), resulting in effective tags. The effective tags were denoised using the DADA2 module in the QIIME2 software (version QIIME2-202202) to generate initial amplicon sequence variants (ASVs). Taxonomic classification was performed using the Silva database (classify-sklearn). Subsequently, analyses of alpha diversity, beta diversity, hierarchical clustering, and MetagenomeSeq were conducted and presented. The genera with |log_2_ fold change (FC)| > 1 and nominal *P* values < 0.05 were initially considered as candidate different. These *P* values were adjusted using the Benjamini–Hochberg procedure to control the false discovery rate (FDR). Genera with FDR < 0.05 were considered statistically significant, whereas those meeting only the nominal threshold were considered exploratory candidates.

### 2.6 Untargeted metabolomics

Untargeted metabolomic profiling of vaginal secretions was performed using UHPLC–MS/MS with a Vanquish UHPLC system coupled to an Orbitrap Q Exactive HF mass spectrometer (Thermo Fisher Scientific, Bremen, Germany). Raw data were processed using Compound Discoverer software (version 3.3, Thermo Fisher Scientific), and data acquired in positive- and negative-ionization modes were combined for subsequent analysis. Putative metabolite annotations were assigned using the Kyoto Encyclopedia of Genes and Genomes (KEGG) database (https://www.genome.jp/kegg/pathway.html), the Human Metabolome Database (HMDB) (https://hmdb.ca/metabolites), and the LIPID MAPS Structure Database (LMSD) (http://www.lipidmaps.org/). In the absence of authentic chemical standards analyzed under identical conditions, all resulting matches represent putative annotations rather than definitive identifications. This is particularly relevant for structurally related lipids and eicosanoids, as their isomeric forms often share similar accurate masses and MS/MS fragmentation patterns.

Unsupervised principal component analysis (PCA) was used to visualize the overall distribution and variation of the metabolomic profiles, and between-group differences in overall metabolomic composition were evaluated using PERMANOVA with 999 permutations. Subsequently, orthogonal projections to latent structures–discriminant analysis (OPLS–DA) was performed as a supervised approach to explore group discrimination. Model performance was evaluated using R^2^Y, which represents the goodness of fit, and Q^2^, which reflects cross-validated predictive ability. A 1,000-permutation test was further performed to assess model validity and potential overfitting. The contribution of individual metabolite features to supervised group discrimination was evaluated using variable importance in projection (VIP) scores derived from the OPLS-DA model. Candidate differential metabolite features were identified using the predefined thresholds of VIP > 1, |log_2_FC| > 1, and nominal *P* < 0.05. Pathway enrichment and topology analyses were performed using the KEGG database and the signaling pathway impact analysis (SPIA) algorithm (19). Benjamini–Hochberg correction was applied across the tested pathways, and pathways with FDR < 0.05 were considered statistically significant.

Spearman’s rank-order correlation analysis was performed across all samples to assess correlations between the relative abundances of candidate differential genera and the peak intensities of key metabolites. Partial Spearman correlation analysis controlling for the experimental group was conducted to reduce the potential influence of group separation. Correlation coefficients (Spearman’s ρ) and two-sided nominal *P* values were calculated for each genus–metabolite pair. Nominal *P* values were adjusted using the Benjamini–Hochberg method, with FDR-adjusted *q* < 0.05 considered statistically significant. Associations with nominal *P* < 0.05 but FDR-adjusted *q* ≥ 0.05 were considered exploratory.

### 2.7 Western blot

Western blot was applied to analyze the key proteins of the Toll-like receptor (TLR)/nuclear factor-kappa B (NF-κB) inflammatory axis in uterine tissues. Tissues were lysed in RIPA buffer (Beyotime, Shanghai, China) containing 1% protease inhibitor cocktail and phosphatase inhibitor cocktail (MedChemExpress, Monmouth Junction, NJ, USA). Total protein concentrations were determined using a BCA assay kit (Beyotime). Protein samples were mixed with loading buffer and boiled for 5 min. Equal amounts of protein were separated by sodium dodecyl sulfate–polyacrylamide gel electrophoresis (SDS–PAGE) and transferred onto PVDF membranes. After blocking with a rapid blocking buffer (Epizyme, Shanghai, China), the blots were incubated overnight at 4 °C with the following primary antibodies: TLR2 (Abcam, Cambridge, UK; dilution 1:1000), TLR4 (Proteintech, Hubei, China; dilution 1:1000), phospho-NF-κB p65 at serine 536 (pS536-p65; Proteintech; dilution 1:2000), cyclooxygenase-2 (COX-2; Proteintech; dilution 1:1000), and glyceraldehyde-3-phosphate dehydrogenase (GAPDH; Proteintech; dilution 1:10000). The next day, the membranes were incubated with HRP-conjugated secondary antibodies (Proteintech; dilution 1:10000) for 1 h at room temperature (**Supplementary Table 2**). Immunoreactive bands were visualized using an ECL substrate (Bio-Rad, Hercules, CA, USA). Signal intensities were quantified via Image Lab software (Bio-Rad). The band intensities of TLR2, TLR4, pS536-p65, and COX-2 were individually normalized to their corresponding GAPDH loading controls from the same membrane. Total p65 was unmeasured; therefore, a pS536-p65/total p65 ratio was unavailable. Western blotting was performed using three independent uterine tissue samples per group.

### 2.8 Statistical analysis

The normality and homogeneity of the data were assessed by the Shapiro-Wilk test and the Levene test, respectively. Data that followed a normal distribution and had homogeneous variances were analyzed with two-sided unpaired Student’s *t-tests*. Numerical results in the text and tables are reported as means (x̄) ± standard deviations (SD). Error bars in **Figures 1E–F**, **4B**, and **5A** represent the standard error of the mean (SEM). Unless otherwise specified, *P* values < 0.05 were considered statistically significant.

## 3 Result

### 3.1 Characterization of the *Gardnerella vaginalis*-associated bacterial vaginosis-like rat model

The observation of “clue cells” (*G. vaginalis* biofilm-coated epithelial cells) by vaginal Gram staining is a characteristic diagnostic feature of BV (8). In the model group, clue cell-like epithelial cells were observed in the vaginal smears, whereas no bacteria were detected on the epithelial cells in the control group (**Figure 1B**). Gross examination of the excised reproductive tracts revealed that the model group had increased vaginal discharge and noticeable edema of the vagina and uterus. In contrast, the control group showed a normal structure (**Figure 1C**). H&E staining demonstrated that in the model group, the vaginal tissues exhibited significant damage characterized by epithelial cell exfoliation and inflammatory cell infiltration in the mucosa and lamina propria. The uterine cavity was dilated, with disruption of the uterine wall and inflammatory infiltration in both the mucosal and muscular layers. Conversely, the control group displayed intact vaginal and endometrial epithelial cells (**Figure 1D**). Additionally, the quantitative analysis of vaginal stratified squamous epithelium thickness revealed a significant increase in the model group compared to the control group (*P* = 0.001; **Supplementary Table 3**; **Figure 1E**). The serum levels of IL-1*β*, IL-6, TNF-*α*, and TGF-*β* were elevated, whereas IL-10 was reduced in the model group (all *P* < 0.001; **Supplementary Table 4**; **Figure 1F**). Together, these findings indicated that antibiotic pretreatment followed by repeated intravaginal *G. vaginalis* inoculation established a rat model exhibiting multiple BV-relevant features.

### 3.2 Dysregulation of vaginal microbiota in rats with *Gardnerella vaginalis*-associated bacterial vaginosis

The descriptive Venn diagram showed that 479 ASVs were shared between the two groups, whereas 1,315 and 795 ASVs were detected only in the model and control groups, respectively (**Figure 2A**). Alpha diversity analyses were performed to evaluate within-sample microbial diversity. Although differences were not statistically significant (all *P* > 0.05), the model group showed an increasing trend in both the Shannon and Dominance indices and a decreasing trend in Pielou’s evenness compared to the control group (**Supplementary Table 5**; **Figure 2B**). Beta diversity analyses were applied to compare the composition of microbial communities between groups. The principal coordinate analysis (PCoA) based on Bray–Curtis distances at the ASV level showed visual differences in the distribution of model and control samples, with the first principal coordinate (PC1) and the second principal coordinate (PC2) explaining 43.5% and 19.3% of the total variation, respectively. However, the PERMANOVA did not identify a statistically significant difference in overall microbial community composition between the two groups (Adonis, R^2^ = 0.12, *P* = 0.061; **Figure 2C**). Permutational analysis of multivariate dispersion (PERMDISP) based on 999 permutations revealed significantly greater within-group multivariate dispersion in the control group than in the model group (*F* = 4.72, *P* = 0.039; **Figure 2D**), with mean distances to the group centroids of 0.53 and 0.42, respectively.

**Figure 2:**
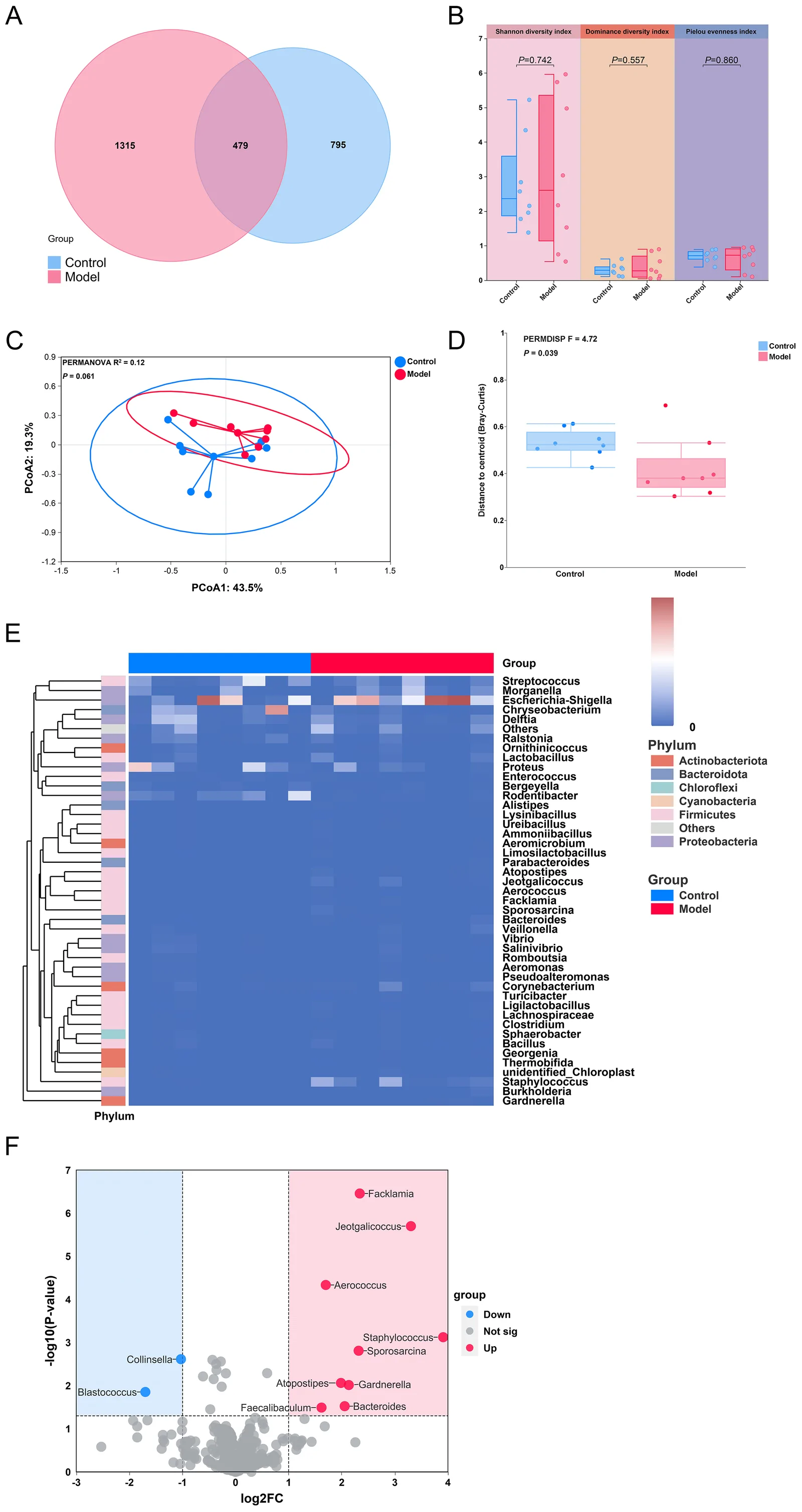
Dysregulation of vaginal microbiota in rats with *Gardnerella vaginalis*-associated bacterial vaginosis. **(A)** Distribution of amplicon sequence variants (ASVs) between the control group and the model group. The Venn diagram summarizes shared and group-specific ASVs and is not intended as a statistical test of community differentiation. **(B)** Alpha diversity represented by the Shannon diversity index, the Dominance index, and the Pielou’s evenness index. Data are shown as box plots with individual data points; n = 8 rats per group. Statistical significance for alpha-diversity comparisons was assessed using Student’s *t*-tests. **(C)** Principal coordinate analysis (PCoA) based on Bray–Curtis distances at the ASV level, used to visualize the distribution of individual samples according to overall microbial community composition. Differences in group centroids were assessed using permutational multivariate analysis of variance (PERMANOVA) via Adonis (R^2^ = 0.12, *P* = 0.061). **(D)** Homogeneity of within-group multivariate dispersion was evaluated separately using permutational analysis of multivariate dispersion (PERMDISP) with 999 permutations. The mean distances to the group centroids were 0.53 and 0.42 for the control and model groups, respectively (*F* = 4.72, *P* = 0.039). **(E)** Hierarchical clustering heatmap showing sample-level abundance patterns of the 45 most abundant genera, representing 6 phyla, across the control and model groups. Relative abundances were log-transformed and subsequently standardized within each genus using row-wise Z scores. Hierarchical clustering was performed using Euclidean distance and complete linkage. The annotation on the left indicates the phylum-level classification of each genus, and the annotation at the top indicates the experimental group of each sample. **(F)** Volcano plot of genus-level differential-abundance analysis using MetagenomeSeq. Genera with |log_2_ fold change (FC)| > 1 and nominal *P* < 0.05 were initially identified as candidate differential genera. The *P* values were adjusted using the Benjamini–Hochberg procedure to control the false discovery rate (FDR); genera with FDR < 0.05 were considered statistically significant, whereas the remaining candidate genera were considered exploratory. Complete differential-abundance results are provided in **Supplementary Table 6**.

The hierarchical clustering heatmap analysis, using Euclidean distances and complete linkage clustering, visualized abundance patterns of the top 45 genera belonging to 6 phyla across individual samples (**Figure 2E**). MetagenomeSeq analysis identified 11 candidate differential genera meeting the initial thresholds of |log_2_FC| > 1 and nominal *P* < 0.05, including 9 enriched and 2 depleted genera in the model group. After FDR correction, only *Jeotgalicoccus* (log_2_FC = 3.30, FDR < 0.001), *Facklamia* (log_2_FC = 2.34, FDR < 0.001), and *Aerococcus* (log_2_FC = 1.70, FDR = 0.008) remained significantly enriched. Among the genera highlighted in the subsequent integrative analysis, *Staphylococcus* (log_2_FC = 3.91), *Gardnerella* (log_2_FC = 2.13), and *Bacteroides* (log_2_FC = 2.06) showed positive effect estimates, whereas *Collinsella* showed a negative effect estimate (log_2_FC = −1.03). These differences did not remain significant after FDR correction and were therefore considered exploratory findings (**Supplementary Table 6**; **Figure 2F**).

### 3.3 Alteration of vaginal metabolic features in rats with *Gardnerella vaginalis*-associated bacterial vaginosis

A total of 806 metabolites were detected across the combined positive- and negative-ionization datasets. Unsupervised PCA, which utilized Euclidean distance, showed partial visual separation between the model and control groups, with PC1 and PC2 together explaining 49% of the total variance. However, the difference in overall metabolomic composition was not statistically significant by PERMANOVA (R^2^ = 0.15, *P* = 0.138; **Figure 3A**). OPLS-DA was subsequently applied as a supervised approach to evaluate group discrimination. The OPLS-DA model demonstrated an R^2^Y of 77.3% and a cumulative Q^2^ of 45.8%, indicating good model fit and moderate cross-validated predictive ability. The corresponding 1,000-permutation test further supported the validity of the supervised model and confirmed the absence of overfitting (*P* < 0.001; **Figure 3B**). Based on the predefined exploratory thresholds of VIP > 1, |log_2_FC| > 1, and nominal *P* < 0.05, 111 candidate differential metabolite features were detected, including 36 increased and 75 decreased features in the model group (**Figure 3C**).

**Figure 3:**
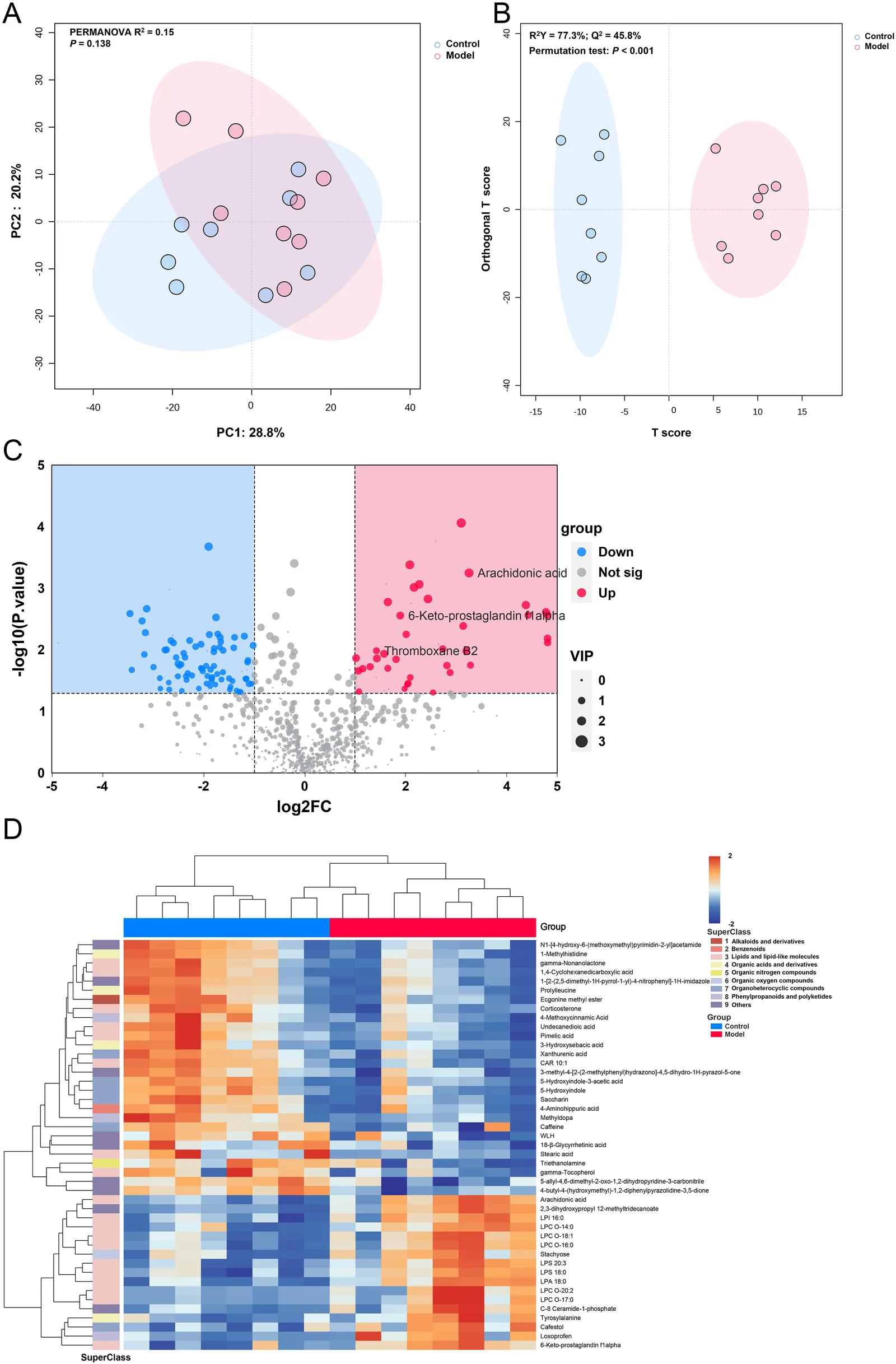
Alteration of vaginal metabolic features in rats with *Gardnerella vaginalis*-associated bacterial vaginosis. **(A)** Principal component analysis (PCA) based on Euclidean distance, used to visualize the distribution of individual samples according to overall metabolomic composition. Differences in overall metabolomic composition were assessed using permutational multivariate analysis of variance (PERMANOVA) with 999 permutations (R^2^ = 0.15, *P* = 0.138). **(B)** Orthogonal projections to latent structures–discriminant analysis (OPLS-DA) and corresponding 1,000-permutation test. The OPLS-DA model demonstrated an R^2^Y of 77.3% and a cumulative Q^2^ of 45.8%. The permutation test supported model validity and confirmed the absence of overfitting (*P* < 0.001). **(C)** Volcano plot of vaginal metabolic features. A total of 111 candidate differential metabolite features were identified using variable importance in projection (VIP) > 1, |log_2_ fold change (FC)| > 1, and nominal *P* < 0.05, including 36 increased and 75 decreased features in the model group. **(D)** Hierarchical clustering heatmap showing sample-level intensity patterns of the top 45 candidate differential metabolite features across the control and model groups. Relative intensities were log-transformed and subsequently standardized within each metabolite feature using row-wise Z scores. Hierarchical clustering was performed using Euclidean distance and complete linkage. The annotation at the top indicates the experimental group of each sample. Complete candidate differential metabolite results are provided in **Supplementary Table 7**.

These candidate features were predominantly assigned to lipids and lipid-like molecules. Several putatively annotated glycerophospholipid-derived lysophospholipids were increased in the model group, including LPA 18:0, LPI 16:0, lysophosphatidylserines (Lyso-PS) species (lysoPS 20:3 and lysoPS 18:0), and ether-linked LPC species (LPC O-14:0, LPC O-16:0, and LPC O-18:1). These changes may reflect enhanced membrane phospholipid remodeling or hydrolysis. A feature putatively annotated as C-8 ceramide-1-phosphate (C8-C1P) was also increased. In contrast, several features associated with fatty acid oxidation (FAO) were decreased, represented by CAR 10:1, 3-hydroxysebacic acid, pimelic acid, and undecanedioic acid (**Supplementary Table 7**; **Figure 3D**).

### 3.4 Upregulation of arachidonic acid metabolism and compartment-specific remodeling of tryptophan metabolism in rats with *Gardnerella vaginalis*-associated bacterial vaginosis

Metabolic pathway analysis of the candidate differential metabolite features detected in vaginal secretions identified 11 significantly altered pathways after correction for multiple testing (FDR < 0.05; **Supplementary Table 8**). Among these pathways, AA metabolism and TRP metabolism were prioritized for further analysis. AA metabolism exhibited the highest pathway impact value (impact value = 0.30, FDR = 0.036) and included increased vaginal features putatively annotated as AA (VIP = 2.23, log_2_ FC = 3.26, *P* < 0.001) and the AA-derived eicosanoids 6-keto-PGF1_α_ (VIP = 1.89, log_2_ FC = 1.90, *P* = 0.003) and TXB_2_ (VIP = 1.64, log_2_ FC = 1.42, *P* = 0.010), supporting enhanced AA-related metabolic activity in the vaginal compartment. TRP metabolism was also significantly altered at the pathway level (impact value = 0.03, FDR = 0.036) and included decreased vaginal features putatively annotated as xanthurenic acid (XA; VIP = 1.82, log_2_ FC = -3.12, P = 0.002), 5-hydroxyindole-3-acetic acid (5-HIAA; VIP = 1.45, log_2_ FC = -1.68, P = 0.009), and KYNA (VIP = 1.39, log_2_ FC = -1.79, P = 0.018; **Figure 4A**).

**Figure 4:**
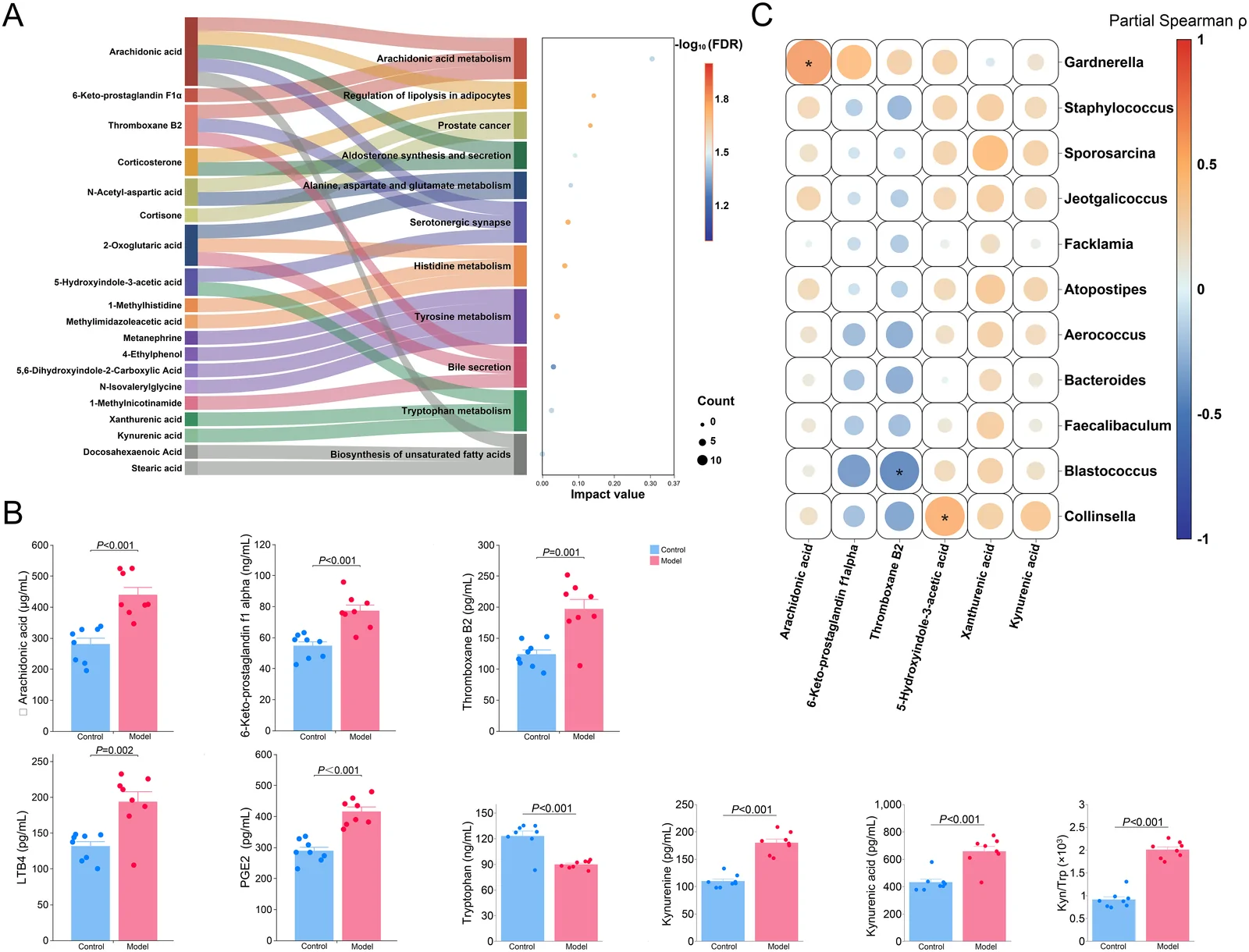
Upregulation of arachidonic acid metabolism and compartment-specific remodeling of tryptophan metabolism in rats with *Gardnerella vaginalis*-associated bacterial vaginosis. **(A)** Pathway enrichment analysis of candidate differential metabolite features identified in vaginal secretions. Metabolic pathways were identified using the Kyoto Encyclopedia of Genes and Genomes (KEGG) database and signaling pathway impact analysis (SPIA) algorithm. Benjamini–Hochberg correction was applied across tested pathways; pathways with false discovery rate (FDR) < 0.05 were considered statistically significant. Complete pathway enrichment results are provided in **Supplementary Table 8**. **(B)** Serum concentrations of arachidonic acid (AA), 6-keto-prostaglandin F1 alpha (6-keto-PGF_1α_), thromboxane B_2_ (TXB_2_), leukotriene B_4_ (LTB_4_), prostaglandin E_2_ (PGE_2_), tryptophan (TRP), kynurenine (KYN), and kynurenic acid (KYNA), together with the KYN-to-TRP ratio (KYN/TRP) reported on a ×10^3^ scale. Data are presented as the means ( $\bar{X}$) ± standard error of the mean (SEM); n = 8 rats per group. Statistical significance was determined using Student’s *t-tests*. Complete serum metabolite results are provided in **Supplementary Table 9**. **(C)** Bubble correlation matrix of partial Spearman correlation coefficients for associations between candidate differential genera and key metabolites involved in arachidonic acid and tryptophan metabolism after adjustment for experimental group. Bubble size represents the absolute value of the partial correlation coefficient, whereas bubble color indicates the direction and magnitude of the association. Asterisks indicate nominal *P* < 0.05; none of the associations remained statistically significant after Benjamini–Hochberg false discovery rate (FDR) correction (all *q* > 0.05). Complete correlation results are provided in **Supplementary Table 10**.

Targeted serum measurements showed that free AA, 6-keto-PGF_1α_, TXB_2_, LTB_4_ and PGE_2_ concentrations were significantly higher in the model group (all *P* ≤ 0.002). However, serum TRP was decreased, whereas KYN, KYNA and the KYN/TRP ratio were increased in the model group (all *P* < 0.001; **Supplementary Table 9**; **Figure 4B**). Notably, the putatively annotated KYNA feature in vaginal secretions was decreased, whereas serum KYNA was increased, suggesting compartment-specific remodeling of TRP metabolism rather than uniform suppression.

To further explore potential microbiota–metabolite links, Spearman correlation analysis was performed between the 11 candidate differential genera and 6 key vaginal metabolites involved in AA and TRP metabolism. Partial Spearman correlation analysis controlling for experimental group was further performed to reduce the potential influence of between-group separation. Nominal positive correlations were observed between *Gardnerella* and AA (partial ρ = 0.64, *P* = 0.010) and between *Collinsella* and 5-HIAA (partial ρ = 0.54, *P* = 0.036), whereas *Blastococcus* was negatively correlated with TXB_2_ (partial ρ = −0.54, *P* = 0.038; **Figure 4C**). However, none of these correlations remained statistically significant after FDR correction (all *q* > 0.05), and the findings should therefore be considered exploratory (**Supplementary Table 10**).

### 3.5 Upregulation of proteins associated with uterine TLR2/4–NF-κB–COX-2 inflammatory signaling in rats with *Gardnerella vaginalis*-associated bacterial vaginosis

Western blot analysis showed that uterine TLR2, TLR4, and COX-2 levels normalized to GAPDH were significantly higher in the model group than in the controls. The abundance of pS536-p65 normalized to GAPDH was also significantly increased (all *P* < 0.05; **Supplementary Table 11**, **Figures 5A, B**). Total p65 was not measured; therefore, a pS536-p65/total p65 ratio was not available. These results demonstrate coordinated increases in proteins related to uterine TLR2/4–NF-κB–COX-2 inflammatory signaling.

**Figure 5:**
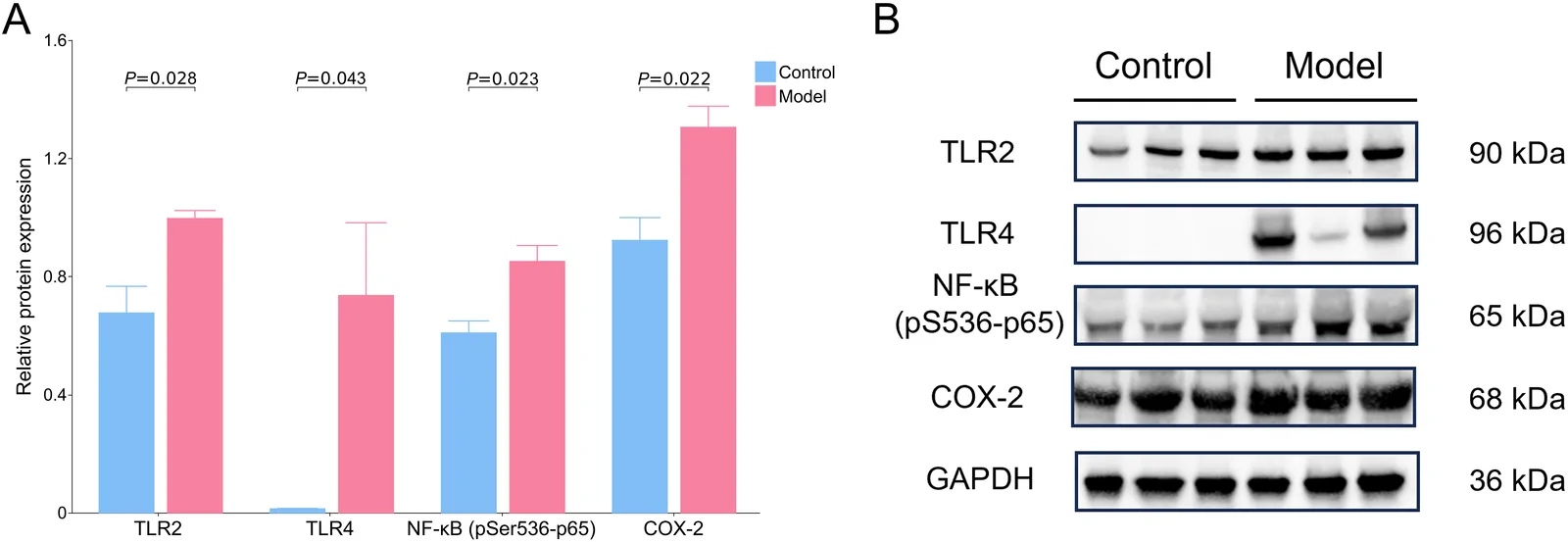
Upregulation of proteins associated with uterine TLR2/4–NF-κB–COX-2 inflammatory signaling in rats with *Gardnerella vaginalis*-associated bacterial vaginosis. **(A)** Relative protein abundance of Toll-like receptor 2 (TLR2), TLR4, phospho-NF-κB p65 at serine 536 (pS536-p65) and cyclooxygenase-2 (COX-2) in uterine tissues. Data are presented as the means ( $\bar{X}$) ± standard error of the mean (SEM); n = 3 independent uterine tissue samples per group. Statistical significance was determined using Student’s *t-tests*. Complete densitometric values are provided in **Supplementary Table 11**. **(B)** Representative Western blot images. Protein levels were normalized to glyceraldehyde-3-phosphate dehydrogenase (GAPDH). Original uncropped Western blot images are provided in **Supplementary Figure 1**.

## 4 Discussion

The present study established a *Gardnerella vaginalis*-associated BV-like rat model and integrated multi-omics analyses to systematically characterize the interconnected changes in the vaginal microbiota, metabolome, and host inflammatory and immunomodulatory signaling. The model reproduced several key BV-relevant features, accompanied by uterine inflammation and systemic cytokine imbalance. Integrated analysis revealed vaginal microbial alterations, broad lipid disturbances marked by alterations in putatively annotated AA-related metabolites, compartment-specific remodeling of TRP metabolism, and upregulation of proteins related to the uterine TLR/NF-κB axis. Together, these findings provide a microbiome–metabolome–immune framework for understanding the inflammatory and immunometabolic features of this BV-like phenotype, while generating hypotheses regarding its potential role in endometrial inflammation.

Unlike commonly used short-term colonization protocols involving exogenous estradiol (20–22), we employed an antibiotic pretreatment followed by a 28-day intravaginal *G. vaginalis* exposure regimen without exogenous hormonal intervention. Enrolling rats in diestrus minimized baseline estrous cycle-related variation between groups. Although estradiol pretreatment is frequently used to facilitate bacterial persistence in vaginal models (23), it may also affect epithelial turnover and glycogen availability, potentially confounding the interpretation of downstream microbial, metabolic, and inflammatory changes. The prolonged, estradiol-free design therefore provided a useful platform for characterizing sustained host–microbe interactions while minimizing potential confounding from exogenous hormones. This approach is consistent with previous rat vaginal dysbiosis model. Chen et al. (24) established a rat vaginal dysbiosis model using antibiotic pretreatment and demonstrated that vaginal microbiota transplantation and probiotic combinations efficiently rescued the dysbiotic state. This study supports the utility of antibiotic-pretreated rat models for investigating host–microbe interactions in vaginal dysbiosis. However, because vaginal cytology was not continuously monitored during the modeling period, endogenous estrous-cycle fluctuations remain a potential source of variation in epithelial morphology, microbial composition, and metabolic profiles at the time of sampling.

Following the combined modeling procedure, the model reproduced several BV-relevant features, including clue cell-like epithelial cells and increased vaginal discharge. It also exhibited vaginal epithelial injury with inflammatory cell infiltration, uterine histopathological changes, and an altered serum cytokine profile. Clinical studies have similarly associated *Gardnerella* enrichment and BV-associated microbial states with epithelial barrier dysfunction and mucosal immune perturbations (25, 26). However, the clinical and immunological manifestations of human BV are heterogeneous, and BV does not invariably produce pronounced inflammation (3). The relatively inflammatory phenotype observed here may therefore limit direct extrapolation to asymptomatic or uncomplicated human BV, while providing an experimental platform for investigating relationships among sustained vaginal injury, upper reproductive tract changes, and systemic inflammatory responses.

Using this model, we evaluated the ecological shifts in the vaginal microbiota. The model group exhibited numerically higher Shannon and Dominance indices and a numerically lower Pielou’s evenness index; however, none of these alpha-diversity comparisons reached statistical significance. These findings should therefore be interpreted as numerical trends rather than evidence of significant changes in within-sample diversity or evenness. In particular, an increase in the Dominance index may reflect greater prominence of a subset of taxa. Beta-diversity analysis showed visual differences in sample distribution, but PERMANOVA did not detect a statistically significant difference in overall community composition. The significant PERMDISP result indicated lower within-group dispersion in the model group than in the control group, suggesting a difference in within-group variability that should be considered when interpreting the PERMANOVA result, rather than definitive separation of group centroids. In addition, the group-specific ASVs shown in the Venn diagram should be interpreted descriptively and do not provide independent statistical evidence of community differentiation. At the genus level, *Aerococcus* remained significant after FDR correction. *Gardnerella*, *Staphylococcus*, *Bacteroides*, and *Collinsella* did not remain significant after FDR correction and were therefore retained only as exploratory candidates. These findings identify candidate taxonomic changes following the combined modeling procedure but do not establish broad, uniform dysbiosis or demonstrate that G. vaginalis alone caused the observed microbial alterations.

From a broader clinical perspective, human BV is generally recognized as a heterogeneous, polymicrobial condition involving changes in community composition and ecological organization, including interactions among multiple facultative and obligate anaerobes and biofilm-associated taxa (2, 3, 6, 15, 27). The relevance of vaginal microbial disturbance may extend beyond classical BV. Recent clinical microbiome studies have reported reduced *Lactobacillus* abundance together with changes in *Gardnerella* or other community members in endometriosis (28), as well as increased microbial richness, evenness, network complexity, and dysbiosis-associated taxa accompanied by reduced *Lactobacillus* in postpartum women with stress urinary incontinence (29). Although these disorders differ substantially in pathophysiology and these studies do not establish causality, they support the broader concept that disruption of *Lactobacillus*-associated homeostasis may accompany diverse gynecological or pelvic disorders. However, these human clinical observations should be carefully distinguished from the findings in our rat model. No animal model fully reproduces the anatomical, hormonal, physicochemical, and microbial complexity of the human vagina, and species-specific differences may affect microbial colonization and host responses (30). In particular, rodent vaginal microbial ecology differs from the *Lactobacillus*-dominated communities commonly observed in healthy humans, and experimental *G. vaginalis* inoculation in rats may not recapitulate the polymicrobial dynamics of human BV (31). The present model should therefore be viewed as an experimental platform for investigating host responses linked to vaginal disturbance rather than as a direct reproduction of the human BV microbiome.

With these translational limitations in mind, the taxonomic findings in the present model can be considered in relation to microbial patterns previously described in BV. *Aerococcus* has been reported as a co-occurring microbe in some clinical BV-associated microbial communities (18, 27), although its functional role in the present rat model remains to be established. Similarly, the exploratory increase in *Bacteroides* may be compatible with the expansion of anaerobic taxa within an altered vaginal community, whereas the nominal changes in *Staphylococcus* and *Gardnerella* may reflect candidate alterations in facultative or co-occurring taxa under disturbed mucosal conditions (3, 18, 27). From a translational perspective, the antibiotic pretreatment used in this model also highlights broader concerns that prolonged or broad-spectrum antimicrobial exposure may affect microbial community resilience and ecological function. This consideration supports further investigation of microbiome-preserving or function-targeted interventions. As a proof of principle outside the vaginal field, selective inhibition of bacterial sialidase has been reported to preserve microbial community resilience while enhancing mucosal protection (32). Whether similar function-targeted strategies can be applied to BV remains to be investigated.

Beyond microbial community shifts, the BV-like phenotype was accompanied by epithelial barrier injury and candidate lipid-related metabolic alterations. The histological findings of epithelial exfoliation, inflammatory infiltration, and increased epithelial thickness provided tissue-level evidence of mucosal injury, which is consistent with previous reports that *G. vaginalis* can impair vaginal epithelial barrier function and activate mucosal immune and inflammatory responses (16, 33). At the metabolic level, lipids and lipid-like molecules were the most prominent class assigned among the candidate differential features. Several features putatively annotated as glycerophospholipid-derived lysophospholipids were increased, including LPA 18:0, LPI 16:0, Lyso-PS species such as lysoPS 20:3 and lysoPS 18:0, and ether-linked LPC species including LPC O-14:0, LPC O-16:0, and LPC O-18:1. Lysophospholipids can be generated through membrane phospholipid hydrolysis and may act as bioactive lipid signals involved in inflammatory cell activation, oxidative stress, and innate immune signaling (34–36). Accordingly, the increased abundance of these putatively annotated features may be consistent with enhanced membrane phospholipid remodeling or hydrolysis and associated lipid signaling in the vaginal mucosa. In addition, a feature putatively annotated as C8-C1P was increased, which may be consistent with sphingolipid-related stress signaling, given the role of ceramide-1-phosphate (C1P) as a bioactive sphingolipid mediator of inflammatory responses (37, 38). The reduction of features associated with FAO, including CAR 10:1, 3-hydroxysebacic acid, pimelic acid, and undecanedioic acid, may further suggest altered FAO-related metabolic activity under inflammatory conditions, which may be relevant to immune-cell activation and inflammatory phenotypes (39, 40). Together, these findings suggest that the BV-like phenotype following the combined modeling procedure was accompanied by candidate lipid-related metabolic changes, including patterns consistent with membrane phospholipid remodeling, sphingolipid-related stress signaling, and altered FAO-related activity. However, these interpretations remain exploratory because the overall metabolomic composition did not differ significantly by PERMANOVA and the candidate differential features were selected using nominal statistical thresholds. Moreover, because the metabolite identities were assigned putatively rather than confirmed using authentic standards analyzed under identical conditions, the specific metabolite identities and associated pathway-level interpretations require further validation.

Within the altered lipid landscape, AA metabolism exhibited the highest pathway impact value among the significantly altered pathways, characterized by increased vaginal metabolic features putatively annotated as AA, 6-keto-PGF1_α_, and TXB_2_. This AA-centered pattern is consistent with a specific inflammatory lipid signature within the broader metabolic remodeling described in clinical BV. Recent multi-omics and metabolic-network studies have shown that complex microbial interactions can alter vaginal lipid and fatty acid profiles, including LysoPC(18:1(9Z)) and eicosenoic acid (18, 27). Mechanistically, this inflammatory signature may involve the mobilization of AA, an ω-6 polyunsaturated fatty acid (PUFA) commonly esterified in membrane phospholipids (41). Upon membrane phospholipid hydrolysis, AA is liberated—a process that may be associated with the concurrent increase in the vaginal feature putatively annotated as C8-C1P, as C1P is known to trigger cytosolic phospholipase A2 (cPLA2) activation and subsequent AA mobilization (37, 42). Once released, AA is rapidly metabolized via the cyclooxygenase (COX) and lipoxygenase (LOX) pathways into downstream eicosanoids, including prostaglandins (PGs), thromboxanes (TXs), and leukotrienes (LTs) (43–45). Given the well-established role of these AA-derived eicosanoids as potent inflammatory mediators, we further evaluated whether AA-related alterations were also detectable in serum. Targeted serum measurements showed significantly higher concentrations of free AA, 6-keto-PGF1_α_, TXB_2_, LTB_4_, and PGE_2_ in the model group. These lipid mediators are known to promote vascular permeability, leukocyte recruitment, and smooth muscle contraction (45, 46). The observed AA-related changes may therefore be relevant to vaginal discharge, tissue edema, and inflammatory infiltration observed in the model group. The concordant increases in vaginal features putatively annotated as AA, 6-keto-PGF1_α,_ and TXB_2_ and their corresponding serum measurements support AA-related metabolic alterations across the vaginal and systemic compartments. However, these findings do not establish that vaginal AA-related alterations caused or amplified systemic inflammation. Rather, they suggest a potential metabolic link between the local BV-like phenotype and the broader inflammatory changes observed in this model. Because the vaginal features and serum analytes were assessed using different analytical platforms and biological matrices, the serum measurements should be considered complementary evidence rather than direct validation of the untargeted vaginal metabolite annotations. Targeted LC-MS/MS with isotope-labeled internal standards would be a valuable approach for future independent validation of these small-molecule measurements.

Alongside these AA-related inflammatory lipid alterations, our metabolomic profiling identified a significant pathway-level alteration in TRP metabolism. TRP and its downstream bioactive metabolites are critical regulators of mucosal immune tolerance, tissue homeostasis, and inflammatory regulation, exerting their effects primarily via immunoregulatory pathways such as aryl hydrocarbon receptor (AHR) signaling (47–50). The model group exhibited a decrease in vaginal features putatively annotated as 5-HIAA, XA, and KYNA, which may be consistent with altered TRP-dependent mucosal immune regulation within the vaginal microenvironment. This interpretation is supported indirectly by studies on intestinal inflammation showing that the depletion of TRP-derived metabolites, such as XA and KYNA, can be linked to aggravated inflammatory responses, while their restoration may protect tissues via AHR-related mechanisms (51, 52). Conversely, systemic circulation showed a marked depletion of TRP accompanied by elevated KYN, KYNA, and the KYN/TRP ratio. This serum profile may be consistent with enhanced inflammation-associated TRP catabolism through the KYN pathway. A similar mechanism has been described in gut dysbiosis, in which pathogen-triggered mucosal inflammation upregulates indoleamine 2,3-dioxygenase 1 (IDO1), ultimately leading to circulating TRP depletion and KYN accumulation (53). Taken together, the decrease in vaginal features putatively annotated as TRP-related metabolites, together with decreased serum TRP and increased serum KYN, KYNA, and the KYN/TRP ratio, supports compartment-specific remodeling of TRP metabolism rather than uniform pathway suppression. Notably, the opposite directions of vaginal and serum KYNA further emphasize this spatial heterogeneity. These findings may reflect distinct local and systemic immunometabolic responses associated with the BV-like phenotype, although whether the vaginal and serum alterations arise from the same biological process remains unclear, and this spatial heterogeneity cannot yet be assigned to a specific enzymatic mechanism.

Connecting the observed vaginal microbial changes to these metabolic alterations, our correlation analysis explored potential microbial–metabolic associations involving the AA-related inflammatory profile and compartment-specific remodeling of TRP metabolism. Because correlations across pooled samples could be influenced by between-group separation, partial Spearman correlations controlling for experimental group were used for interpretation. After group adjustment, *Gardnerella* was nominally positively associated with AA, whereas *Collinsella* was nominally positively associated with 5-HIAA and *Blastococcus* was nominally negatively associated with TXB_2_. These multi-omics correlations suggest potential associations between specific vaginal genera and local AA- or TRP-related metabolic features, although they do not establish direct microbial–metabolic interactions. In particular, the nominal *Gardnerella*–AA association may indicate a potentially linked pattern between this BV-associated taxon and the AA-related vaginal metabolic profile, but it does not demonstrate that *Gardnerella* directly induced AA mobilization or metabolism. In contrast, the nominally associated *Collinsella*–5-HIAA pattern may suggest a potential relationship between this genus and local TRP-related metabolism. While direct evidence linking vaginal *Collinsella* to TRP metabolism remains limited, recent gut microbiome–metabolome studies have identified *Collinsella* as a potential microbial contributor to the TRP–indole pathway, where its metabolic outputs can shift between immunoregulatory indole production and pro-inflammatory KYN pathway activation depending on the local microenvironment (54, 55). The *Collinsella*–5-HIAA relationship observed here may therefore represent a hypothesis-generating association, rather than evidence that the depletion of *Collinsella* weakened immunoregulatory metabolic activity. The nominal inverse association between *Blastococcus* and TXB_2_ should be interpreted with similar caution. Importantly, none of these group-adjusted associations remained statistically significant after Benjamini–Hochberg correction across the tested genus–metabolite pairs; therefore, they require independent validation and should not be interpreted as evidence of causal or direct microbial–metabolic interactions.

Building upon these local and systemic immunometabolic disturbances, uterine histological and protein alterations were also observed. The model group exhibited uterine edema, cavity dilation, disruption of the uterine wall, and inflammatory infiltration across both mucosal and muscular layers. At the molecular level, this tissue injury coincided with significantly increased GAPDH-normalized abundance of uterine TLR2, TLR4, pS536-p65, and COX-2. These coordinated changes support the involvement of proteins associated with uterine TLR2/4–NF-κB–COX-2 inflammatory signaling, although they do not establish sequential pathway activation. Evaluating both TLR2 and TLR4 provides a broader assessment of proteins involved in innate immune sensing in this model. While previous studies have identified TLR2 as a primary receptor for *G. vaginalis*-associated mucosal inflammation by sensing its microbial components and triggering epithelial NF-κB signaling (16, 56), clinical BV remains a profoundly polymicrobial condition (1). In our model, the candidate enrichment of co-occurring bacteria such as *Bacteroides*, *Staphylococcus*, and *Aerococcus* may represent a broader array of pathogen-associated molecular patterns (PAMPs). Concurrently, the extensive epithelial injury may release endogenous damage-associated molecular patterns (DAMPs). Together with circulating cytokines and AA-derived eicosanoids, this diverse pool of PAMPs and DAMPs could potentially contribute to uterine innate immune signaling involving both TLR2 and TLR4, which are widely expressed across the reproductive tract (44, 57, 58). In established inflammatory signaling pathways, TLR2/4 engagement can activate NF-κB signaling, which may be linked to AA metabolism through the induction of COX-2. NF-κB-dependent induction of COX-2, a key enzyme converting AA into PGs, is a recognized mechanism for amplifying inflammation within reproductive tissues (44, 58, 59). TRP-related metabolic changes may also be relevant to this inflammatory context. Previous studies have shown that the KYN-pathway metabolites KYNA and XA can participate in AHR-mediated regulation of NF-κB signaling (60) and COX-2 expression (61). Accordingly, the decreased vaginal features putatively annotated as KYNA and XA may be compatible with altered local AHR-related immunoregulation. However, AHR activity was not measured, and these vaginal metabolic changes cannot be assumed to directly regulate inflammatory signaling in uterine tissue. Overall, the coordinated increases in GAPDH-normalized TLR2, TLR4, pS536-p65, and COX-2 abundance support the involvement of uterine TLR2/4–NF-κB–COX-2-related inflammatory signaling. However, these findings do not establish sequential pathway activation or demonstrate that the microbial and metabolic alterations caused the observed protein changes. Pathway inhibition or rescue experiments will be required to determine the functional and causal relationships among these signaling components.

In considering the possible routes linking vaginal disturbance to uterine inflammation, current studies suggest that BV-associated upper reproductive tract complications may occur through mechanisms that do not necessarily require direct bacterial invasion of the uterus, pointing to broader disruption of mucosal and systemic immunity (3, 62). However, neither *G. vaginalis* nor total bacterial burden was assessed in uterine tissue in the present study. Direct ascending microbial exposure therefore cannot be excluded. Accordingly, direct ascending exposure and indirect host signaling through systemic inflammatory or immunometabolic alterations should be considered alternative, non-mutually exclusive explanations for the observed uterine inflammation. Building on this understanding, our findings show that the BV-like phenotype was accompanied by vaginal microbial and metabolic alterations, systemic inflammatory changes, uterine histopathological injury, and increased abundance of proteins associated with uterine TLR2/4–NF-κB–COX-2 inflammatory signaling. These observations support a potential relationship between vaginal disturbance and upper reproductive tract inflammation but do not distinguish between direct ascending microbial exposure and indirect systemic or immunometabolic signaling.

Several limitations in this study should be acknowledged. First, our model combined antibiotic pretreatment with repeated *G. vaginalis* inoculation, whereas the control group received saline alone; therefore, their individual contributions cannot be distinguished. Although the findings support a BV-like phenotype, they should be interpreted as effects of the combined modeling procedure rather than attributed solely to *G. vaginalis*. Future studies should include an antibiotic-pretreated saline control to clarify the specific contribution of *G. vaginalis*. Second, although enrollment was restricted to rats in diestrus, vaginal cytology was not monitored during the prolonged modeling period. Therefore, the effects of endogenous cycling on vaginal epithelial, microbial, and metabolic features could not be excluded. Third, vaginal pH, absolute *G. vaginalis* burden, post-inoculation bacterial culture, and species-specific quantitative PCR were not assessed. These omissions limited the quantitative characterization of the vaginal environment and prevented confirmation of viable *G. vaginalis* persistence at sampling. Future studies should incorporate these measurements to provide more direct evidence of model establishment and colonization dynamics.

## 5 Conclusion

In conclusion, the *G. vaginalis*-associated BV-like phenotype following the combined modeling procedure was accompanied by vaginal microbial alterations, enhanced AA-related metabolism, compartment-specific remodeling of TRP metabolism, and increased abundance of proteins associated with the uterine TLR/NF-κB axis. These observations define a candidate microbiome–metabolome–immune network that may help explain how *G. vaginalis*-associated vaginal disturbance relates to BV-like inflammatory changes and potential upper reproductive tract complications. However, the relationships identified remain primarily associative and cross-sectional, and functional validation studies are warranted to establish causal links and mechanistic pathways.

## Data Availability

The raw 16S rRNA gene sequencing data have been deposited in the Sequence Read Archive (SRA) of the National Center for Biotechnology Information (NCBI) under BioProject accession PRJNA1522624. The untargeted LC–MS metabolomics data have been deposited in MetaboLights under accession MTBLS15608. Summary results are provided in the article and Supplementary Material.
