# Supplementary material for: Inflammatory landscape of Gardnerella vaginalis-associated bacterial vaginosis in rats: an integrated multi-omics analysis
Source: Front Immunol. 2026 Sep 22;17:1914434. doi: 10.3389/fimmu.2026.1914434 (PMC13638667; doi:10.3389/fimmu.2026.1914434)
Supplement: Supplementary file 1 [file DataSheet1.pdf]

Supplementary Table S1.
Characteristics of the ELISA kits used for serum cytokine and metabolite measurements.

Analyte	Catalogue no.	Detection range	Sample dilution	Intra- / inter-assay CV (%)	Manufacturer-reported cross-reactivity
IL-1β	MM-0047R1	1.0 - 480 pg/mL	5-fold	≤15 / ≤15	None reported
IL-6	MM-0190R1	1.0 - 48 pg/mL	5-fold	≤15 / ≤15	None reported
IL-10	MM-0195R1	1.0 - 40 pg/mL	5-fold	≤15 / ≤15	None reported
TNF-α	MM-0180R1	1.0 - 320 pg/mL	5-fold	≤15 / ≤15	None reported
TGF-β	MM-20594R1	1.0 - 800 pg/mL	5-fold	≤15 / ≤15	None reported
Free AA	MM-0929R1	1.0 - 480 μg/mL	5-fold	≤15 / ≤15	None reported
6-keto-$PGF_{1\alpha}$	MM-0268R1	1.0 - 80 ng/mL	5-fold	≤15 / ≤15	None reported
TXB_2	MM-0516R2	1.0 - 240 pg/mL	5-fold	≤15 / ≤15	None reported
LTB_4	MM-0048R1	1.0 - 240 pg/mL	5-fold	≤15 / ≤15	None reported
PGE_2	MM-0068R1	5.0 - 480 pg/mL	5-fold	≤15 / ≤15	None reported
TRP	MM-926267O1	1.0 - 160 ng/mL	5-fold	≤15 / ≤15	None reported
KYN	MM-928072O1	1.0 - 800 pg/mL	5-fold	≤15 / ≤15	None reported
KYNA	MM-928848O1	1.0 - 200 pg/mL	5-fold	≤15 / ≤15	None reported

All ELISA kits were purchased from Jiangsu Meimian Industrial Co., Ltd. (Jiangsu, China). ELISA, enzyme-linked immunosorbent assay; IL-1β, interleukin-1 beta; TNF-α, tumor necrosis factor-alpha; TGF-β, transforming growth factor-beta; AA, arachidonic acid; 6-keto-$PGF_{1\alpha}$, 6-keto-prostaglandin F1 alpha; TXB_2, thromboxane B_2; LTB_4, leukotriene B_4; PGE_2, prostaglandin E_2; TRP, tryptophan; KYN, kynurenine; KYNA, kynurenic acid.

Supplementary Table S2.
Characteristics of the antibodies used for western blot analysis.

Target protein	Host species	Manufacturer	Catalogue no.	Dilution
TLR2	Rabbit	Abcam	ab209217	1:1000
TLR4	Mouse	Proteintech	66350-1-IG	1:1000
pS536-p65	Rabbit	Proteintech	80379-2-RR	1:2000
COX-2	Mouse	Proteintech	66351-1-IG	1:1000
GAPDH	Mouse	Proteintech	60004-1-Ig	1:10000
HRP-conjugated Goat Anti-Mouse IgG (H+L)	Goat	Proteintech	SA00001-1	1:10000
HRP-conjugated Goat Anti-Rabbit IgG (H+L)	Goat	Proteintech	SA00001-2	1:10000

TLR2, toll-like receptor 2; pS536-p65, phospho-NF-κB p65 at serine 536; COX-2, cyclooxygenase-2; GAPDH, glyceraldehyde-3-phosphate dehydrogenase.

Supplementary Table S3.
Quantitative analysis of stratified squamous epithelium thickness.

Stratified squamous epithelium thickness (μm)	Control (N = 8)	Model (N = 8)	*P*-value
	53.8 ± 11.9 (43.9, 63.7)	106.5 ± 35.2 (77.1, 135.8)	0.001

Data are presented as the means ($\bar{X}$) ± standard deviations (SD) and 95% confidence interval (CI). Statistical significance was determined using Student's *t-tests*.

Supplementary Table S4.
Concentrations of serum cytokines.

Cytokine	Control (N = 8)	Model (N = 8)	*P*-value
IL-1β (pg/mL)	250.9 ± 51.3 (208.1, 293.8)	574.9 ± 62.8 (522.4, 627.5)	< 0.001
IL-6 (pg/mL)	23.4 ± 3.9 (20.1, 26.6)	52.3 ± 4.8 (48.3, 56.2)	< 0.001
TNF-α (pg/mL)	184.7 ± 31.1 (158.7, 210.7)	349.4 ± 30.2 (324.2, 374.7)	< 0.001
TGF-β (pg/mL)	443.2 ± 73.3 (381.9, 504.5)	863.0 ± 74.7 (800.6, 925.5)	< 0.001
IL-10 (pg/mL)	37.3 ± 7.4 (31.1, 43.4)	16.3 ± 3.1 (13.7, 18.9)	< 0.001

Data are presented as the means ($\bar{X}$) ± standard deviations (SD) and 95% confidence interval (CI). Statistical significance was determined using Student's *t-tests*. IL-1β, interleukin-1 beta; TNF-α, tumor necrosis factor-alpha; TGF-β, transforming growth factor-beta.

Supplementary Table S5.
Alpha diversity indices.

Index	Control (N = 8)	Model (N = 8)	*P*-value
Shannon diversity	2.78 ± 1.34 (1.66, 3.90)	3.09 ± 2.21 (1.24, 4.93)	0.742
Dominance	0.30 ± 0.17 (0.16, 0.44)	0.38 ± 0.34 (0.10, 0.67)	0.557
Pielou's evenness	0.38 ± 0.11 (0.28, 0.47)	0.36 ± 0.22 (0.18, 0.54)	0.860

Data are presented as the means ($\bar{X}$) ± standard deviations (SD) and 95% confidence interval (CI). Statistical significance was determined using Student's *t-tests*.

Supplementary Table S6.

Candidate differential genera identified by MetagenomeSeq using |log₂FC| > 1 and nominal *P* < 0.05.

Genera	log_2FC	*P*-value	FDR
Staphylococcus	3.91	< 0.001	0.092
Jeotgalicoccus	3.30	< 0.001	< 0.001
Facklamia	2.34	< 0.001	< 0.001
Sporosarcina	2.32	0.002	0.152
Gardnerella	2.13	0.010	0.300
Bacteroides	2.06	0.030	0.701
Atopostipes	1.99	0.009	0.282
Aerococcus	1.70	< 0.001	0.008
Faecalibaculum	1.62	0.032	0.701
Blastococcus	-1.70	0.014	0.381
Collinsella	-1.03	0.002	0.168

Candidate differential genera were identified using MetagenomeSeq with the initial screening thresholds of $|log_2FC| > 1$ and nominal $P < 0.05$. Statistical significance after multiple-testing correction was defined as FDR < 0.05. FC, fold change; FDR, false discovery rate.

Supplementary Table S7.
Putatively annotated candidate differential metabolites identified by untargeted metabolomics using VIP > 1, $|log_2FC| > 1$, and nominal *P* < 0.05.

Metabolites	Super Class	VIP	log_2FC	*P*-value
LPC O-20:2	Lipids and lipid-like molecules	1.75	4.81	0.008
LPC O-17:0	Lipids and lipid-like molecules	1.81	4.81	0.007
C-8 Ceramide-1-phosphate	Lipids and lipid-like molecules	1.91	4.81	0.003
LPC O-18:1	Lipids and lipid-like molecules	1.98	4.78	0.002
Loxoprofen	Benzenoids	1.72	4.43	0.003
LPC O-16:0	Lipids and lipid-like molecules	2.02	4.38	0.002
2-(2-amino-3-methylbutanamido)-3-phenylpropanoic acid	Organic acids and derivatives	1.64	3.28	0.018
Arachidonic acid	Lipids and lipid-like molecules	2.23	3.26	0.001
Methionine sulfoxide	Organic acids and derivatives	1.82	3.20	0.010
Stachyose	Organic oxygen compounds	1.86	3.14	0.004
2,3-dihydroxypropyl 12-methyltridecanoate	Lipids and lipid-like molecules	2.34	3.10	< 0.001
LPE O-18:1	Lipids and lipid-like molecules	1.63	2.88	0.023

2-methoxy-4-{3-[(4-methylbenzoyl)amino]butyl}phenyl 4-methylbenzoate	Benzenoids	1.76	2.82	0.018
Tyrosylalanine	Organic acids and derivatives	1.71	2.73	0.010
2-Benzyl-5-[(3S)-1-isopropyl-3-pyrrolidinyl]-1,3,4-oxadiazole	Organoheterocyclic compounds	1.37	2.54	0.049
LPI 16:0	Lipids and lipid-like molecules	2.13	2.44	0.001
LPC O-14:0	Lipids and lipid-like molecules	2.12	2.27	0.001
LPS 20:3	Lipids and lipid-like molecules	2.14	2.17	0.001
Alanyltyrosine	Organic acids and derivatives	1.52	2.09	0.028
LPA 18:0	Lipids and lipid-like molecules	2.18	2.08	< 0.001
N-Acetyl-aspartic acid	Organic acids and derivatives	1.64	2.05	0.035
Gly-Tyr-Ala	Organic acids and derivatives	1.51	2.04	0.036
Cafestol	Lipids and lipid-like molecules	1.76	2.01	0.006
Maltotetraose	Organic oxygen compounds	1.35	1.98	0.043
6-Keto-prostaglandin f1alpha	Lipids and lipid-like molecules	1.89	1.90	0.003
Docosahexaenoic Acid	Lipids and lipid-like molecules	1.76	1.81	0.014
LPS 18:0	Lipids and lipid-like molecules	2.04	1.65	0.002
Prostaglandin E1	Lipids and lipid-like molecules	1.63	1.65	0.020
Lysopc 17:0	Lipids and lipid-like molecules	1.90	1.58	0.012
LPC 18:0	Lipids and lipid-like molecules	1.88	1.43	0.014
Thromboxane B2	Lipids and lipid-like molecules	1.64	1.42	0.010
Traumatic acid	Lipids and lipid-like molecules	1.74	1.30	0.019
LysoPE 18:0	Lipids and lipid-like molecules	1.77	1.15	0.020
PC O-20:4	Lipids and lipid-like molecules	1.47	1.08	0.048
LPC 16:0	Lipids and lipid-like molecules	1.78	1.07	0.022
LPC 16:0-SN1	Lipids and lipid-like molecules	1.85	1.02	0.014
Undecanedioic acid	Lipids and lipid-like molecules	1.62	-1.02	0.008
N2,N2-Dimethylguanosine	Organoheterocyclic compounds	1.21	-1.05	0.036
4-(acetylamino)phenyl 3-chlorobenzoate	Benzenoids	1.56	-1.10	0.036
Metanephrine	Organic acids and derivatives	1.54	-1.12	0.015
Azelaic acid	Lipids and lipid-like molecules	1.52	-1.13	0.016
Pantothenic acid	Organic acids and derivatives	1.23	-1.15	0.029
2-Isopropylmalic acid	Lipids and lipid-like molecules	1.37	-1.16	0.030

N-Acetylvaline	Organic acids and derivatives	1.35	-1.17	0.029
Pimelic acid	Lipids and lipid-like molecules	1.64	-1.19	0.006
2-Oxoglutaric acid	Organic acids and derivatives	1.31	-1.27	0.048
N-Isovalerylglycine	Organic acids and derivatives	1.22	-1.30	0.048
7-Methylguanine	Organoheterocyclic compounds	1.24	-1.34	0.046
5-Hydroxyindole	Organoheterocyclic compounds	1.59	-1.38	0.006
Stearic acid	Lipids and lipid-like molecules	1.55	-1.40	0.009
3,8,9-trihydroxy-10-propyl-3,4,5,8,9,10-hexahydro-2H-oxecin-2-one	Organoheterocyclic compounds	1.27	-1.42	0.032
2-Hydroxyhippuric acid	Organic acids and derivatives	1.26	-1.46	0.040
4-Pyridoxic acid	Organoheterocyclic compounds	1.25	-1.48	0.044
N-α-L-Acetyl-arginine	Organic acids and derivatives	1.43	-1.49	0.029
2-Isopropylmalate	Lipids and lipid-like molecules	1.55	-1.61	0.010
7-Hydroxy-3,4-dihydrocarbostyril	Organoheterocyclic compounds	1.37	-1.62	0.023
3-Methyluridine	Organoheterocyclic compounds	1.32	-1.64	0.030
Ecgonine methyl ester	Organoheterocyclic compounds	1.51	-1.66	0.007
3-Hydroxysebacic acid	Lipids and lipid-like molecules	1.66	-1.67	0.006
5-Hydroxyindole-3-acetic acid	Organoheterocyclic compounds	1.45	-1.68	0.009
3-methyl-4-[2-(2-methylphenyl)hydrazono]-4,5-dihydro-1H-pyrazol-5-one	Organoheterocyclic compounds	1.57	-1.72	0.006
N-Phenylacetylglutamine	Organic acids and derivatives	1.33	-1.73	0.025
Methyldopa	Organic acids and derivatives	1.83	-1.75	0.003
Aflatoxin G2	Organoheterocyclic compounds	1.25	-1.76	0.037
4-Aminohippuric acid	Organic acids and derivatives	1.49	-1.79	0.009
Kynurenic acid	Organoheterocyclic compounds	1.39	-1.79	0.018
Methylimidazoleacetic acid	Organic acids and derivatives	1.55	-1.79	0.011
2'-O-Methyluridine	Organoheterocyclic compounds	1.40	-1.83	0.028
D-Glucuronic acid	Organic oxygen compounds	1.35	-1.83	0.029
Dodecanedioic acid	Lipids and lipid-like molecules	1.36	-1.84	0.032
o-Cresol	Benzenoids	1.45	-1.86	0.021
Cortisone	Lipids and lipid-like molecules	1.23	-1.87	0.039
gamma-Nonanolactone	Lipids and lipid-like molecules	1.66	-1.88	0.006
CAR 10:1	Lipids and lipid-like molecules	2.06	-1.90	< 0.001

5,6-Dihydroxyindole-2-Carboxylic Acid	Organoheterocyclic compounds	1.35	-1.92	0.027
Creatinine	Organic acids and derivatives	1.30	-1.94	0.035
Quinoline	Organoheterocyclic compounds	1.38	-1.95	0.018
N-α-Acetyl-L-asparagine	Organic acids and derivatives	1.42	-2.01	0.022
Allantoin	Organoheterocyclic compounds	1.42	-2.03	0.020
Ferulic acid	Benzenoids	1.41	-2.05	0.020
4-Methoxycinnamic Acid	Benzenoids	1.62	-2.05	0.008
Phenylacetylglycine	Organic acids and derivatives	1.23	-2.09	0.042
1,4-Cyclohexanedicarboxylic acid	Lipids and lipid-like molecules	1.57	-2.15	0.009
N-Acetyl-L-carnosine	Organic acids and derivatives	1.42	-2.23	0.026
Prolylleucine	Organic acids and derivatives	1.61	-2.27	0.007
N1-(4-chlorophenyl)-2-cyano-4,4-dimethyl-3-oxopentanamide	Benzenoids	1.43	-2.31	0.024
Tetranor-PGDM	Lipids and lipid-like molecules	1.35	-2.33	0.031
N-Acetyl-L-glutamine	Organic acids and derivatives	1.38	-2.35	0.027
P-Aminohippuric Acid	Organic acids and derivatives	1.51	-2.36	0.013
Capryloylglycine	Organic acids and derivatives	1.50	-2.39	0.011
3-phenyl-5-(trifluoromethyl)-4,5-dihydro-1H-pyrazol-5-ol	Organoheterocyclic compounds	1.20	-2.43	0.046
N-Acetyl-L-histidine	Organic acids and derivatives	1.46	-2.44	0.017
4-Ethylphenol	Benzenoids	1.36	-2.47	0.018
1,3-Dimethyluracil	Organoheterocyclic compounds	1.64	-2.50	0.017
4-Hydroxy-3-methoxyphenylglycol sulfate	Benzenoids	1.35	-2.51	0.027
L-Ascorbic acid 2-sulfate	Organic oxygen compounds	1.47	-2.54	0.013
1-[2-(2,5-dimethyl-1H-pyrrol-1-yl)-4-nitrophenyl]-1H-imidazole	Organoheterocyclic compounds	1.59	-2.59	0.008
N1-[4-hydroxy-6-(methoxymethyl)pyrimidin-2-yl]acetamide	Organoheterocyclic compounds	1.64	-2.64	0.007
VQH	Organic acids and derivatives	1.30	-2.68	0.044
(3-Methoxy-4-hydroxyphenyl)ethylene glycol sulfate	Benzenoids	1.20	-2.69	0.034
6-Methylnicotinamide	Organoheterocyclic compounds	1.62	-2.73	0.010
1-Methylnicotinamide	Organoheterocyclic compounds	1.62	-2.76	0.010
Hippuric acid	Organic acids and derivatives	1.23	-2.85	0.043
trans-Aconitic acid	Organic acids and derivatives	1.31	-2.86	0.029

Nicotinuric acid	Organoheterocyclic compounds	1.42	-2.99	0.019
Xanthurenic acid	Organoheterocyclic compounds	1.82	-3.12	0.002
1-Methylhistidine	Organic acids and derivatives	1.69	-3.15	0.005
5-Sulfosalicylic acid	Benzenoids	1.51	-3.17	0.012
Saccharin	Organoheterocyclic compounds	1.71	-3.21	0.003
SM 8:1;2O/34:1	Lipids and lipid-like molecules	1.44	-3.42	0.021
Corticosterone	Lipids and lipid-like molecules	1.71	-3.45	0.003

VIP, variable importance in projection; FC, fold change.

Supplementary Table S8.
Significantly altered metabolic pathways identified by SPIA using candidate differential metabolite features (FDR < 0.05).

Pathway	Metabolites	Impact value	FDR
Arachidonic acid metabolism	Arachidonic acid; 6-Keto-prostaglandin $F1_{\alpha}$; Thromboxane B_2	0.30	0.036
Regulation of lipolysis in adipocytes	Arachidonic acid; Corticosterone	0.14	0.019
Prostate cancer	N-Acetyl-aspartic acid; Cortisone	0.13	0.019
Aldosterone synthesis and secretion	Arachidonic acid; Corticosterone	0.09	0.033
Alanine, aspartate and glutamate metabolism	N-Acetyl-aspartic acid; 2-Oxoglutaric acid	0.08	0.036
Serotonergic synapse	Arachidonic acid; Thromboxane B_2; 5-Hydroxyindole-3-acetic acid	0.07	0.019
Histidine metabolism	1-Methylhistidine; Methylimidazoleacetic acid; 2-Oxoglutaric acid	0.06	0.019
Tyrosine metabolism	Metanephrine; 4-Ethylphenol; 5,6-Dihydroxyindole-2-Carboxylic Acid; N-Isovalerylglycine	0.04	0.019
Bile secretion	Thromboxane B_2; 1-Methylnicotinamide; 6-Methylnicotinamide; 2-Oxoglutaric acid	0.03	0.048

Tryptophan metabolism	Xanthurenic acid;<br>5-Hydroxyindole-3-acetic acid;<br>Kynurenic acid	0.03	0.036
Biosynthesis of unsaturated fatty acids	Arachidonic acid;<br>Docosahexaenoic Acid;<br>Stearic acid	0.00	0.036

Nominal *P* values were adjusted using the Benjamini–Hochberg method, with FDR-adjusted $q < 0.05$ considered statistically significant. SPIA, signaling pathway impact analysis; FDR, false discovery rate.

Supplementary Table S9.
Concentrations of serum metabolites related to arachidonic acid and tryptophan metabolism.

**Metabolite**	**Control (N = 8)**	**Model (N = 8)**	***P*-value**
Free AA (μg/mL)	279.9 ± 56.7<br>(232.4, 327.3)	438.8 ± 69.9<br>(380.4, 497.2)	< 0.001
6-keto-$PGF_{1\alpha}$ (ng/mL)	54.5 ± 7.7<br>(48.1, 61.0)	77.1 ± 10.9<br>(68.0, 86.2)	< 0.001
TXB_2 (pg/mL)	123.4 ± 21.1<br>(105.8, 141.0)	196.4 ± 45.0<br>(158.8, 234.1)	0.001
LTB_4 (pg/mL)	131.2 ± 19.1<br>(115.3, 147.2)	193.4 ± 40.7<br>(159.3, 227.4)	0.002
PGE_2 (pg/mL)	288.1 ± 35.0<br>(258.9, 317.4)	414.9 ± 44.2<br>(377.9, 451.9)	< 0.001
TRP (ng/mL)	122.9 ± 17.0<br>(108.7, 137.1)	89.7 ± 4.5<br>(85.9, 93.4)	< 0.001
KYN (pg/mL)	109.6 ± 11.7<br>(99.8, 119.4)	179.8 ± 19.4<br>(163.6, 196.0)	< 0.001
KYNA (pg/mL)	429.0 ± 64.9<br>(374.7, 483.2)	657.0 ± 105.5<br>(568.7, 745.2)	< 0.001
KYN/TRP ($\times 10^3$)	0.91 ± 0.18<br>(0.76, 1.06)	2.00 ± 0.18<br>(1.85, 2.15)	< 0.001

Data are presented as the means ($\bar{X}$) ± standard deviations (SD) and 95% confidence interval (CI). Statistical significance was determined using Student's *t-tests*. AA, arachidonic acid; 6-keto-$PGF_{1\alpha}$, 6-keto-prostaglandin F1 alpha; TXB_2, thromboxane B_2; LTB_4, leukotriene B_4; PGE_2, prostaglandin E_2; TRP, tryptophan; KYN, kynurenine; KYNA, kynurenic acid.

Supplementary Table S10.
See "Supplementary Table S10. Pooled Spearman and group-adjusted partial Spearman correlations.xlsx" for details.

Supplementary Table S11.
Relative densitometric values of uterine TLR/NF-κB axis.

Protein	Control (N = 3)	Model (N = 3)	*P*-value
TLR2/GAPDH	0.68 ± 0.16	1.00 ± 0.05	0.028
TLR4/GAPDH	0.013 ± 0.003	0.74 ± 0.43	0.043
pS536-p65/GAPDH	0.61 ± 0.07	0.85 ± 0.09	0.023
COX-2/GAPDH	0.92 ± 0.13	1.31 ± 0.12	0.022

Data are presented as the means ($\bar{X}$) ± standard deviations (SD). Protein signals were normalized to GAPDH. Three independent uterine tissue samples were analyzed per group. Statistical significance was determined using Student's *t-tests*. NF-κB, nuclear factor-kappa B; TLR2, Toll-like receptor 2; pS536-p65, phospho-NF-κB p65 at serine 536; COX-2, cyclooxygenase-2; GAPDH, glyceraldehyde-3-phosphate dehydrogenase.

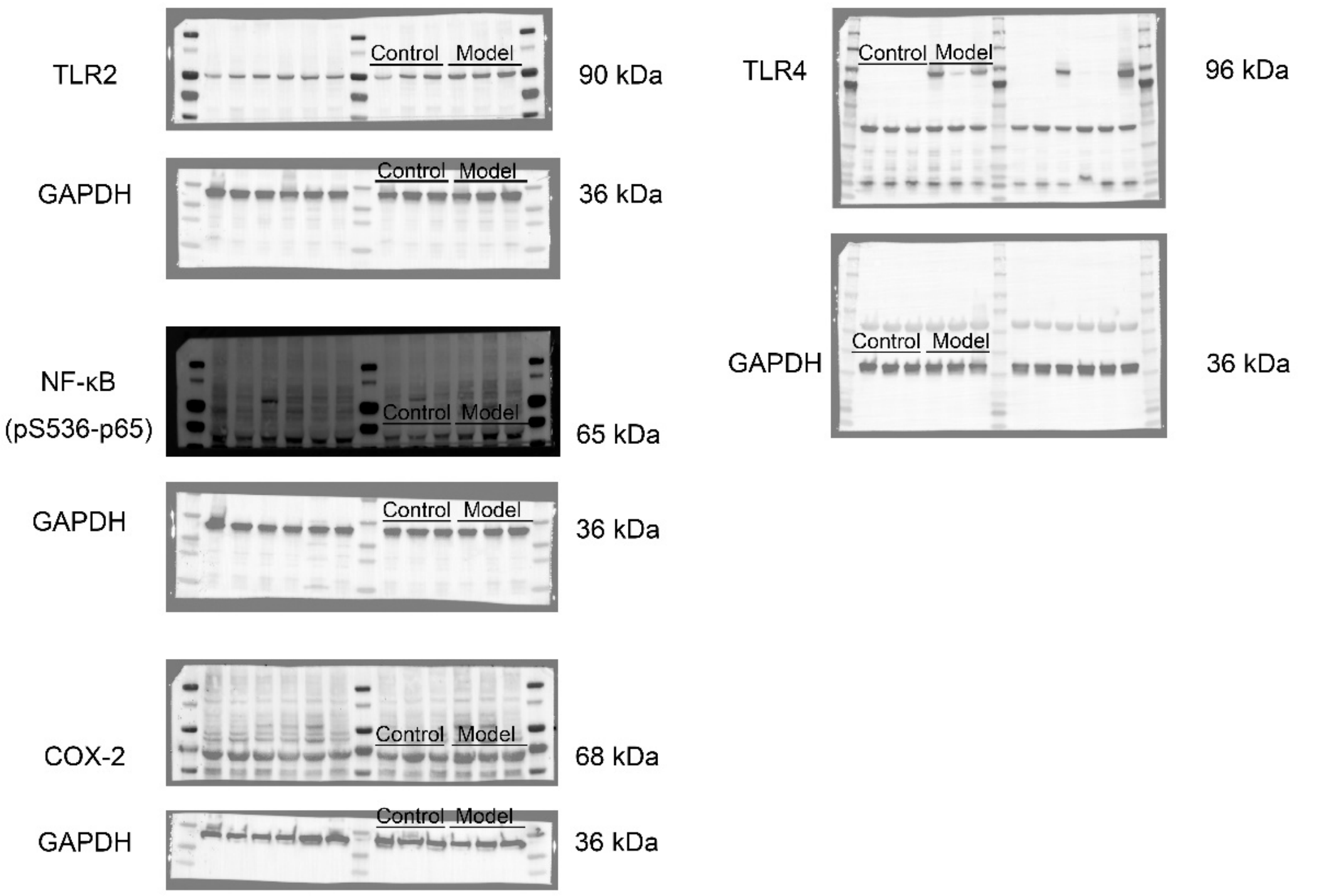

Supplementary Figure S1. Original Western blot images.
